# Hope and depression in Brazilian head and neck cancer patients during the COVID-19 pandemic

**DOI:** 10.3332/ecancer.2022.1371

**Published:** 2022-04-07

**Authors:** Mercedes Nohely Rodriguez Torrealba, Nen Nalú Alves das Mercês, Jorge Vinícius Cestari Felix, Marcio Roberto Paes, Deny Kelson Vasques Pereira, Silvia Francine Sartor

**Affiliations:** 1 Physician, Master’s Degree in Nursing, Federal University of Paraná, Graduate Nursing Programme, Av Professor Lothário Meissner 632, Curitiba PR 80210-170, Brazil; 2 Department of Nursing at the Federal University of Paraná, Av Professor Lothário Meissner 632, Curitiba, PR Paraná 80210-170, Brazil; 3 Head and Neck Department, Erasto Gaertner Hospital, Rua Dr Ovande do Amaral, 201, Jardim das Américas, Curitiba, Paraná 81520-060, Brazil; 4 Graduate Nursing Programme, Federal University of Paraná, Av Professor Lothário Meissner 632, Curitiba PR 80210-170, Brazil

**Keywords:** head and neck neoplasia, hope, depression, coronavirus infections

## Abstract

**Introduction:**

Head and neck cancer is characterised as traumatic, when compared to other types of cancer, due to the physical, physiological and social impact it has on the patient.

**Objective:**

To evaluate hope and severe depression in patients with head and neck cancer during the COVID-19 pandemic.

**Method:**

Quantitative, observational, and analytical; conducted in the outpatient department of the head and neck department of an oncological hospital in the city of Curitiba, Paraná, Brazil, with 60 patients with head and neck cancer being treated with chemotherapy and radiotherapy. Data collection took place between May and September 2020, with the application of three questionnaires: socio-demographic and clinical; Patient Health Questionnaire-9 (PHQ-9), to evaluate severe depression; and the Herth hope scale. Data analysis was through descriptive statistics and non-parametric Mann– Whitney, Kruskal–Wallis and Spearman correlation tests.

**Results:**

The age of participants ranged from 34 to 85 years, with 61.7% being male. The diagnosis of cancer occurred in the oral cavity (53.3%) and larynx (33.3%); 28.3% (*n* = 1 7) presented with a diagnosis of depression; 8.6% (*n* = 5) are in treatment with psychology; and 10.3% (*n* = 6) are in treatment with psychotropic drugs. The analysis of the association of the PHQ-9 score with sex showed a mean score of 7.7 ± 6.2, with a higher level of depression in women. The mean hope score was 41.3 ± 3.1; however, during the pandemic, 35% (*n* = 21) reported feelings of anguish, anxiety and fear, with the latter being predominant and in association with the PHQ-9 score showing a mean of 8.2 ± 6.2 (*p* = 0.123). The association of hope with the number of children was statistically significant (*p* = 0.034) and in the education variable with the PHQ-9 score (*p* = 0.019).

**Conclusion:**

The use of tools that assess both levels of hope and depression in patients undergoing chemotherapy and radiotherapy provides health professionals with support for the implementation of targeted actions to cope with the disease.

## Introduction

Head and neck cancer is the seventh most common cancer worldwide and the eighth leading cause of cancer deaths [1]. Tumours of the lips, oral cavity, pharynx, larynx, nasal cavity and thyroid are grouped as head and neck cancer, which represent the second highest incidence in Brazilian men. Each year, in the triennium 2020–2022, the expected incidence of oral cavity cancer will be 11,180 cases in men and 4,010 in women, and 6,470 in men and 1,180 in women for laryngeal cancer [2]. With regard to mortality, high rates are observed in patients with oral cavity and lip cancer [3].

In December 2019, the first cases of infection from the new coronavirus were registered, called severe acute respiratory syndrome coronavirus 2 (SARS-CoV-2) in China. This illness called coronavirus disease 2019 (COVID-19) disseminated rapidly, turning into a pandemic. Global data reported 172,242,495 confirmed cases of COVID-19 and 3,709,397 deaths by June 2021, and in Brazil there were 16,803,472 confirmed cases and 469,388 deaths were recorded [4].

In that sense, the risk of mortality in patients with head and neck cancer increased by 40% in high-incidence areas in 2020, due to COVID-19. Patients with cancer had a worse clinical outcome compared to other patients with the COVID-19 infection [5].

In the wake of the global health emergency that impacted the activities of cancer hospitals, countries consequently adopted various protocols for the management of COVID-19-infected cancer patients. It was observed that oncology patients are three times more likely to get infected, especially those over 60 years of age, with the risk of severe viral infection increasing fivefold and the risk of death increasing eightfold in cancer patients [6]. According to a study conducted in China, severe complications from COVID-19 are greater in patients with cancer, compared to those without comorbidities [7].

Considering the implications of the diagnosis and treatment of head and neck cancer, associated with the difficulties brought about by the COVID-19 pandemic, the impact of these factors on people’s quality of life is evident in both physical and emotional dimensions [8]. A survey analysed the psychological state of 77 cancer patients during the current COVID-19 pandemic, using the Hospital Anxiety and Depression Scale (HADS) questionnaire, finding depression in 31% of the participants [9].

Therefore, cancer patients, as with the susceptible population, tend to suffer more emotional disorganisation in the face of a public health crisis [10]. COVID-19 collapsed the capacity of healthcare systems around the world, producing a significant impact on cancer patients in terms of diagnostics, treatment and mortality, and especially in the emotional aspect [11]. With the uncertainties of the SARS-CoV-2 pandemic and a diagnosis of head and neck cancer, the possible mental health consequences bring about delays in treatment. It is noteworthy that, during the disease process, psychological states can change significantly, mainly because 35% of them suffer from symptoms of depression and anxiety [12].

The additional complications of the pandemic with restrictions of movement and social isolation may also increase depression in patients with head and neck cancer [13]. Furthermore, the delay in cancer treatment may put additional pressure on the mental health and quality of life of these patients. It is essential to help them maintain their quality of life during this pandemic in order to not affect their general survival outcomes [14].

Depression is a psychological disorder that can be debilitating, potentially lethal and, in severe cases, can lead to suicide [15]. Patients with major depressive disorders experience dysfunctional emotional states and cognitive decline, causing treatment, social and functional problems [16]. Patients with head and neck cancer have a greater risk of emotional stress than patients with other types of cancer. They undergo changes in swallowing, sense of smell, breathing, speaking and, in the worst cases, facial disfigurement during treatment and over the course of the illness [17]. These patients with major depressive symptoms have a low survival rate, with a greater risk of interruptions during cancer treatment and worse responses to the treatment [18]. Therefore, therapy for depression can improve mood, decrease symptoms and improve the prognosis pertaining to the cancer [19].

In this context, hope has a direct impact on physical and psychological well-being, from a higher tolerance to stress to changes in lifestyle and immunological function [20]. Thus, hope allows the cancer patient to live with greater intensity, even in the face of adversities imposed by the illness’ evolution and treatment [21].

In that regard, this study’s objective is to evaluate hope and severe depression in patients with head and neck cancer, during the COVID-19 pandemic.

In this vein, the present article will reveal the results with respect to the aforementioned objective, as well as an association of obtained variables, in addition to analysing the importance of hope in confronting head and neck cancer and its influence on the decrease of depressive symptoms.

## Method

This is a transverse quantitative, observational, analytical study carried out in a designated philanthropic cancer hospital in the state of Paraná, Brazil. The participants were 60 patients diagnosed with head and neck cancer, assisted in outpatient consultation by the head and neck service during the months of May and September in 2020.

The inclusion criteria for the participants were a diagnosis of head and neck cancer of any histopathological kind, in studies T1 through T4 (a or b), submitting to chemotherapy and/or radiation therapy, having retained oral communication or possessing conditions of non-verbal communication through gestures demonstrating the alternative for self-report, indicating the score that expresses how one feels and in the variables evaluated on the scale of depression and hope. The exclusion criteria for participants were auditory deficiency that precludes listening and both verbal and non-verbal communication and an incapacity for the comprehension which would permit verbal or non-verbal communication.

Data collection occurred in a single stage. Three mechanisms were employed: the first for socio-demographic and clinical information which reflects personal and housing data, as well as diagnosis, stage and a pandemic-related question about how these patients were feeling and facing the COVID-19 worldwide health emergency. The second mechanism was the Patient Health Questionnaire-9 (PHQ-9), which evaluates the symptoms of major depressive disorder (MDD) and identifies the risk of developing depression. MDD is defined as a mood disorder characterised by the presence of four or more depressive symptoms like change in mood, lack of interest in doing things, trouble sleeping, fatigue or lack of energy, loss of appetite or weight and feelings of guilt or uselessness, including difficulty concentrating, feeling worried and suicidal thoughts. The presence of symptoms in the last 2 weeks was evaluated on the Likert scale with a score of 0 (nothing or none), 1 (some days), 2 (more than half the days) and 3 (almost every day). The scores range from 0 to 27 points [22].

PHQ-9 scores of 5, 10, 15 and 20 represent light, moderate, moderately severe and severe, respectively [23]. This mechanism contains a tenth question about the symptoms that interfere in the professional, personal or social life of the patient. This question is not a part of the nine former items; its purpose is to rule out normal grief, a history of manic episodes (bipolar disorder) or a physical disorder, medication or other drug as a biological cause of the depressive symptoms [24].

Subsequently, the third mechanism, the Herth hope index, consists of 12 items and evaluates the patients’ stages of hope, in addition to highlighting 3 components of hope: temporality and future, positive expectations and interconnection. It is, therefore, a useful tool [25]. The items’ ratings are established using the Likert scale of 4 points, which range from completely agree to completely disagree, with 1 = completely disagree; 2 = disagree; 3 = agree; and 4 = completely agree. The total score varied from 12 to 48 points; the greater the score, the greater the level of hope [26].

Consequently, the collected data were entered into Microsoft Office Excel® spread sheets. The information was synthesised through statistical analysis. This began with the results from the socio-demographic and clinical questionnaire by means of descriptive analysis, using means, standard deviations, medians and minimum and maximum values for the quantitative variables. For the categorical variables, frequencies and percentages were presented. Subsequently, a comparison between two groups was made, in relation to the scores from PHQ-9 and Herth Hope Index (HHI), using the non-parametric Mann–Whitney test. For comparisons of more than two groups, the non-parametric Kruskal–Wallis test was used; the association between two quantitative variables was analysed by estimating the Spearman’s correlating coefficients. A *p*-value < 0.05 indicated statistical significance. The data were analysed with the Statistical Package for the Social Sciences (SPSS) information programme (International Business Machines Corporation) v.20.0.

This investigation was approved by the Research Ethics Committee for the study’s hospital setting in November of 2019, approved through Ruling No. 3,954,865.

## Results

The 60 participants were aged between 34 and 80 years. The frequencies of the socio-demographic variables are shown in Table 1. It is noted that 61.7% (*n* = 37) were male, 31.7% (*n* = 22) were married and 38.3% (*n* = 23) said they had 1 or 2 children. With respect to the level of education, 43.3% (*n* = 26) did not complete primary school, 78.3% were identified as Catholic and 71.1% were retired. With respect to the clinical data presented in Table 2, 71.7% (*n* = 43) said they had no history of depression; on the other hand, there was a variation in the localisation of the cancer of the head and neck: 53.3% (*n* =32) in the oral cavity (mouth, tongue, mouth floor, retro-molar trigone and alveolar ridge); 33.3% (*n* = 20) larynx; 33.3% (*n* = 20) in oropharynx; 5.0% (*n* = 3) in nasopharynx; and 8.3% (*n* = 5) maxillary adenoids and maxillary bone with reference to the size of the associated tumour (T) and lymphatic involvement (N); it was observed that 16% (*n* = 8) were in stage T2 and 14% (*n* = 7) were in T3.

With reference to the current treatment of depression, 77.6% (*n* = 47) are without treatment; however, 10.3% (*n* = 6) are in treatment with psycho-pharmaceuticals and 8.6% (*n* = 5) are in treatment with psychotherapy. According to the current cancer treatment, 6.7% (*n* = 4) are receiving treatment with chemotherapy and radiotherapy; 30.0% (*n* = 18) with chemotherapy, radiotherapy and surgery; 5.0% (*n* = 3) with chemotherapy and surgery; and 58.3% (*n* = 35) with surgery and radiotherapy.

In this study, the participants expressed feelings regarding the situation of the COVID-19 pandemic. Of the 60 participants, 5% (*n* = 3) expressed being calm, around 31.6% (*n* = 18) expressed fear and 61% (*n* = 35) mentioned their belief in God and having hope. It seems that, in addition to the situation of coping with cancer and its treatment, for some patients the pandemic has caused greater fear.

In Table 3, the descriptive data of the two scores highlight the PHQ-9 with a mean of 6.4 and the Escala de Esperança de Herth (EEH) with a mean of 41.3.

**Figure 1.**
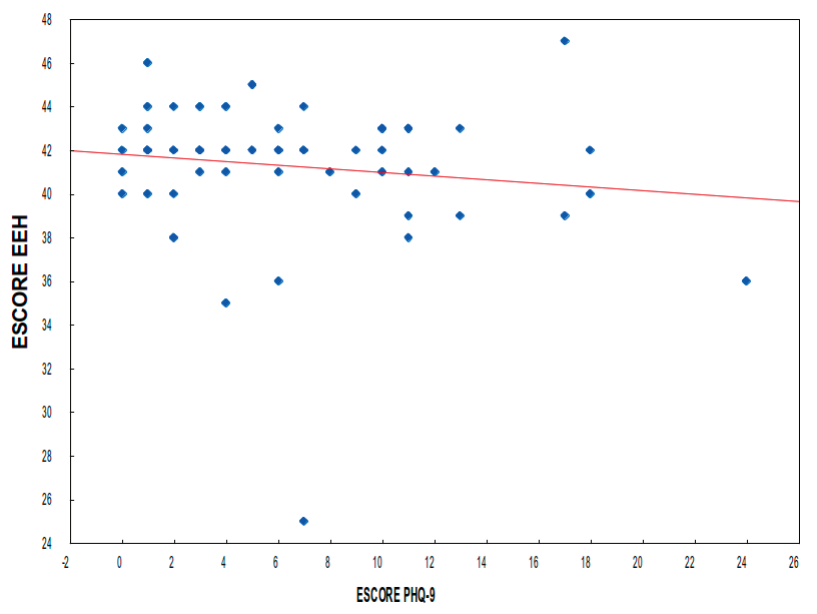
Correlation between EEH and PHQ-9 scores. Dispersion of the two scores, with each point of the graph corresponding to each of the participants.

The correlation between the EEH and PHQ-9 scores is shown in Figure 1. The estimated correlation coefficient was equal to −0.14 without statistical significance (*p* = 0.275).

In this context, for each of the scores and for each of the categorical variables analysed, it was observed that there is no statistically significant correlation between the variables, with the scores through the non-parametric Kruskal–Wallis test with a value of *p* < 0.05.

Next, data on the evaluation of the association between the variable number of children with the PHQ-9 and EHH scores are presented, showing statistical significance between the relationship of expectancy and the number of children. This result highlights that patients who have three or more children presented a higher level of hope (*p* = 0.034). The schooling variable with the association of the PHQ-9 score showed higher levels of depression, statistically significant, in patients with a higher educational level (*p* = 0.019). The fear variable as a feeling expressed during the pandemic, in association with the PHQ-9 scores, showed mild depression (*p* = 0.276), and the cancer diagnosis variable, according to its location with the PHQ-9 association, showed moderate depression in patients diagnosed with cancer in the oropharynx. A mean value plus standard deviation of 10 ± 7.5 was obtained in association with cancer in the oral cavity, larynx, pharynx and oropharynx with the EHH score (*p* = 0.597), thus showing greater hope than depression. In the same way, the association of cancer treatment with the EHH score (*p* = 0.363) determined a higher level of hope.

## Discussion

The present study identified symptoms of major depression among Brazilian patients with head and neck cancer during the COVID-19 pandemic, highlighting a higher level of depression in women. However, it was not statistically significant.

In relation to the socio-demographic variables of our study, the age range of the participants with head and neck cancer was from 34 to 80 years, and they were mostly male. A recent investigation evaluated the influence of socio-demographic and clinical variables in patients with head and neck cancer, highlighting in its results that 60.6% were male patients [27]. Similarly, a cross-sectional study of 69 patients showed that 72.1% were male [28].

The incidence of head and neck cancer in the last decade has increased. Data from 133 patients with head and neck cancer were analysed at an oncology reference centre in southern Brazil. The results showed prevalence in men (65.4%) older than 50 years. In relation to the stage (TNM), 30.8% were found in stage T2 and 20.6% in T3, while 26.32% presented with cancer in the oral cavity and 24.06% in the oropharynx [29].

In our study, a low educational level was observed, and the clinical profile showed cancer in the oral cavity and larynx at stages T2 and T3, respectively. According to the National Cancer Institute records, 16.7% of the cases are found in the oral cavity, 8.8% in the larynx and 44.2% in the facial skin. However, tumour location may vary depending on the country [30].

In relation to religion, it was possible to observe Catholic and evangelical patients. They were mostly single, widowed, divorced and retired with low salary levels and in treatment with radiotherapy and surgery. On another note, the patients in our study reported feelings in relation to the COVID-19 pandemic, including expressions of fear, anxiety and anguish. In this context, we are currently living in uncertain times, and the COVID-19 pandemic continues to cause damage worldwide. One study says that the pandemic affects the quality of life of oncology patients [31]. Another study applied the natural language processing technique and artificial intelligence algorithms in the framework of the Primary Care Evaluation of Mental Disorders to explore the main problems of cancer patients during the COVID-19 pandemic and analyse the emotional burden. A set of learning algorithms was utilised to learn the patients’ positive and negative feelings. The results show eight emotions and feelings such as anger, fear, anticipation, confidence, surprise, sadness, joy and disgust. They also show a significant negative feeling, with fear being the predominant emotion [32]. In our study, the fear variable was associated with the PHQ-9, which detected mild depression in the participants, concluding that negative feelings could influence the development of depression.

It was also found that the participants had a history of depression. However, a study conducted in the Netherlands with 345 patients diagnosed with head and neck cancer confirmed the increased risk of depression before treatment in 15.1% of the patients [33]. Cancer patients often present with depression, which makes it difficult to recognise their presenting set of symptoms [34]. The PHQ-9 is a standard screening instrument for depressive disorders in cancer patients [35].

In the present study, the results of the PHQ-9 showed mild depression in the participants, observing a higher level of depression in women. In this vein, it is necessary to highlight the significance between the education variable with the PHQ-9 because education is characterised as an important factor that makes understanding possible and facilitates the communication and understanding of cancer patients [36]. Patients with a higher level of education presented greater depression, thus determining that education facilitates the ability to understand the diagnosis and its risks.

On the other hand, hope interferes with people in times of crisis, a fact observed in a study of 92 cancer patients. When the Herth hope index-PT was applied, the levels of hope were high, with a mean of 29.61 points [37].

According to the responses in the HERT-Brasil in our study, hope obtained a mean of 41.3. It is possible to observe high levels of hope. This study highlights the association of the children variable with the EHH score (*p* = 0.034), concluding that patients who have more than three children present with greater hope. A cross-sectional study in China noted that the number of children was associated with a lower prevalence of depression in women. Children represent a stronger social support that provides better mental health outcomes [38]. Hope can be nurtured, maintained and strengthened, so positive relationships with family and friends, emotional, social and family support are needed. Hope is one of the foundations of a dignified and comforting death [39].

Cancer patients experience major changes that generate emotional suffering, and hope helps to resolve the difficulties that arise. In a non-Brazilian cross-sectional study conducted using the HADS and the Herth hope scale, high levels of hope, with a mean of 39.6, and levels of depression, with a mean and standard deviation of 5.82 ± 4.12, were obtained in 118 cancer patients, suggesting that hope may protect people from depressive symptoms [40].

Therefore, in the present study, the correlation of the PQH-9 and EEH scores was analysed (*p* = 0.275). However, higher levels of hope were observed, with an average of 41.3, and lower levels of depression, with an average of 6.4, determined as higher level of hope, and lower level of depression.

Cancer patients may feel hopeless, and hopelessness is a symptom of clinical depression, and these patients are three times more likely to commit suicide than the rest of the population [41]. For this reason, in recent years, there has been increased interest in studies on hope, considering the experience of hope in cancer patients to be important [42].

On the other hand, in the present study, a lower percentage of participants had a history of depression, and a higher percentage denied a history of depression, some of them emphasised that they were under treatment with psychotropic drugs and psychotherapy. A study of 321 patients with advanced cancer revealed the great importance of psychotherapy focused on individual meaning. A comparison was made between supportive psychotherapy and improving usual care, the patient experiencing psychological distress, such as depression, and the importance of psychotherapeutic treatment in improving quality of life and reducing suffering [43].

Finally, moderate depression was observed in patients undergoing psychotherapy treatment. Considering the percentages of depression in oncology patients, the importance of psychotherapy and pharmacological treatment and strategies for coping with depression in health services is highlighted. A review study suggesting that the approach of pharmacological treatment together with psychotherapeutic treatment in patients with high levels of depression is ideal determined that selective serotonin reuptake inhibitors are effective for the treatment of depression in cancer patients [44].

In relation to oncological treatment, it will depend on the location of the tumour, the stage of the disease and the feasibility of the therapeutic approaches to be implemented. There are three approaches for advanced cancer: platinum-based chemo-radiotherapy, with surgery reserved for residual disease, surgery with neck resection and reconstruction; followed by adjuvant radiotherapy or chemo-radiotherapy, depending on the presence of adverse risk factors; and induction chemotherapy, followed by definitive chemo-radiotherapy and surgery [45]. In our study, most of the participants are undergoing current treatment with surgery plus radiotherapy.

In this order of ideas, the association of the PQH-9 score with the anatomical location of the tumour determined a greater depressive symptom in patients diagnosed with oropharyngeal cancer. Treatment in patients generates quality of life side effects related to functional changes in saliva production and feeding problems. In addition, alterations in speech, smell and aesthetics, which generate great emotional fragility and impact on quality of life, compromise occupational and social aspects [46]. In the present study, greater depression was obtained in the group of participants treated with chemotherapy and radiotherapy.

## Conclusion

Most of the patients did not present symptoms of major depression, with all the main factors associated with this condition during the COVID-19 pandemic, such as social isolation that generated greater emotional overload due to uncertainty in addition to the risk of exposure when receiving treatment, triggering emotional imbalance and manifesting negative feelings, such as fear, anguish and anxiety.

It was confirmed that people with head and neck cancer undergoing cancer treatment are more hopeful than depressed during the COVID-19 pandemic. The scores were correlated with socio-demographic data, and considering the aspects addressed, high levels of hope and low levels of depression were observed in these patients. The study revealed that patients with children had a higher level of hope, which highlights the influence of an inner strength mediated by love, and also showed that people with higher levels of education have greater depression, due to the understanding of the diagnosis and the risks it triggers.

The present study can be relevant to improve the practice of health professionals involved in the care of cancer patients, highlighting the importance of assessing depression and hope, in order to promote well-being in coping with the disease, and likewise prevent depressive symptoms. Psychological assistance is essential in oncology patients. This is a key element for their recovery and will allow better management of emotions and coping with stress. The oncology patient experiences physical, emotional and spiritual changes during the process of diagnosis and treatment, making this a painful transition, where feelings such as hope and family support encourage positive scenarios that encourage the patient to move forward and adapt to the disease process. Therefore, it is our duty as health professionals to unite as a whole, facilitating multidisciplinary support, providing a comprehensive approach with a biopsychosocial vision involving personal, family, environmental and spiritual aspects and ensuring quality of life of the oncology patient. This study confirms that the higher the level of hope, the lower the level of depression.

## Conflicts of interest

The authors declare that they have no conflicts of interest.

## Funding

No funding was received for this article.

## Figures and Tables

**Table 1.** Socio-demographic data of the participants.

Variable	*n*	Classification	*n*	Result^a^ %
Age	60		60.6 ± 10.9 (34–80)	
Sex	60	Female	23	38.3%
		Male	37	61.7%
Skin colour	60	White	59	98.3%
		Black	1	1.7%
Number of children	60		2.0 ± 2.0 (0–8)	
		0	17	28.3%
		1 or 2	23	38.3%
		3 or 4	14	23.3%
		5 to 8	6	10.0%
Schooling	60	Did not attend	2	3.3%
		Incomplete primary	26	43.3%
		Incomplete secondary	15	25.0%
		Complete secondary	12	20.0%
		incomplete superior	2	3.3%
		complete superior	3	5.0%
Schooling (grouped)	60	up to incomplete primary	28	46.7%
		Secondary (incomplete/complete)	27	45.0%
		Superior (incomplete/complete)	5	8.3%
Religion	60	Catholic	47	78.3%
		Evangelical	9	15.0%
		Other	3	5.0%
		Secular	1	1.7%
Marital status	60	Single	19	31.7%
		Married	22	36.7%
		Consensual Union	2	3.3%
		Divorced	10	16.7%
		Widowed	7	11.6%
Marital status (grouped)	60	Single/divorced/ widowed	36	60.0%
		Married/consensual union	24	40.0%
Withdrew from work for treatment	13	No	4	30.8%
		Yes	9	69.2%
Retired	45	No	13	28.9%
		Yes	32	71.1%
Individual monthly salary (R$)	49		1,645 ± 1,249 (600–8,000)	
Family monthly salary (R$)	53		2,193 ± 1,589 (600–8,000)	

a Described by mean ± standard deviation (minimum–maximum) or by percentage of frequency

R$ real, Brazilian currency

**Table 2.** Clinical data of the participants.

Variable	*n*	Classification	*n*	%
History of depression	60	No	43	71.7
		Yes	17	28.3
Current diagnosis of cancer (grouped)	60	Oral cavity	32	53.30
		Larynx	20	33.3
		Oropharynx	3	5.0
		Nasopharynx	5	8.3
Current treatment of depression	58	None	45	77.6
		Psychotherapy	5	8.6
		Psycho-pharmaceuticals	6	10.3
		Psychotherapy and Psycho-pharmaceuticals	2	3.4
Current treatment of cancer	60	Chemotherapy + radiotherapy	4	6.7
		Chemotherapy + radio + surgery	18	30.0
		Chemotherapy + surgery	3	5.0
		Surgery + radiotherapy	35	58.3
		Chemotherapy + radiotherapy	4	6.7

**Table 3.** Distribution of participants according to the phq-9 and EEH scores.

Score	*n*	Mean	Standard deviation	Median	Minimum	Maximum
PHQ-9 score	60	6.4	5.3	5	0	24
EEH score	60	41.3	3.1	42	25	47

**Table 4.** Evaluation of the association of the variables with the phq-9 and EEH scores.

Variable	Number of children	*n*	Mean ± standard deviation	Median (min–max)	*p* ^*^
PHQ-9 score	0	17	6.8 ± 5.4	4 (1–18)	
	1 or 2	23	6.4 ± 5.3	5 (0–17)	0.898
	3 or more	20	6.1 ± 5.5	5.5 (0–24)	
EEH score	0	17	40.2 ± 4.2	42 (25–43)	
	1 or 2	2	41.4 ± 2.6	41 (35–47)	
	3 or more	20	42.1 ± 2.4	42.5 (36–45)	^*^0.034
**Variable**	**Schooling**	***n***	**Mean ± standard deviation**	**Median (min–max)**	*p* ^*^
PHQ-9 score	Incomplete primary	28	6.0 ± 4.4	6 (0–18)	
	Secondary (incomplete/complete)	27	5.6 ± 5.7	4 (0–24)	^*^0.019
	superior (incomplete/complete)	5	13.0 ± 4.3	12 (9–18)	
EEH score	Incomplete primary	28	41.5 ± 2.3	42 (35–46)	
	Secondary (incomplete/complete)	27	41.0 ± 3.8	42 (25–45)	0.923
	superior (incomplete/complete)	5	42.0 ± 2.9	41 (40–47)	
**Variable**	**Fear**	***n***	**Mean ± standard deviation**	**Median (min–max)**	*p* ^*^
PHQ-9 score	No	38	6.1 ± 5.5	5 (0–24)	0.276
	YES	18	7.6 ± 5.4	7.5 (1–18)	
EEH score	No	38	41.2 ± 3.6	42 (25–46)	0.379
	Yes	18	41.3 ± 2.3	41.5 (38–47)	
**Variable**	**Diagnosis**	***n***	**Mean ± standard deviation**	**Median (min–max)**	
PHQ-9 score	Mouth/tongue/oral cavity	32	5.9 ± 4.9	4.5 (0–17)	
	Larynx	20	6.1 ± 4.6	5.5 (0–18)	0.736
	Nasopharynx	5	8.6 ± 9.1	5 (1–24)	
	Oropharynx	3	10 ± 7.5	9 (3–18)	
EEH score	Mouth/tongue/oral cavity	32	40.8 ± 3.7	42 (25–47)	
	Larynx	20	41.9 ± 1.7	42 (38–45)	0.597
	Nasopharynx	5	42.2 ± 4.1	44 (36–46)	
	Oropharynx	3	42 ± 2	42 (40–44)	
**Variable**	**Current treatment**	***n***	**Mean ± standard deviation**	**Median (min–max)**	*p* ^*^
PHQ-9 score	None	45	6.3 ± 5.3	5 (0–24)	
	Psychotherapy	5	12.8 ± 4.3	12 (7–17)	
	Psycho-pharmaceuticals	6	4.0 ± 3.6	2 (1–10)	0.018
	Psychotherapy and Psycho-pharmaceuticals	2	5.0 ± 2.8	5 (3–7)	
EEH score	None	45	41.5 ± 2.3	42 (35–46)	
	Psychotherapy	5	42.8 ± 3.0	43 (39–47)	0.266
	Psycho-pharmaceuticals	6	38.0 ± 6.7	40 (25–43)	
	Psychotherapy and Psycho-pharmaceuticals	2	42 ± 0		
**Variable**	**Current treatment**	***n***	**Mean ± standard deviation**	**Median (min–max)**	
PHQ-9 score	Radiotherapy + surgery	35	6.4 ± 5.7	5 (0–24)	
	Chemotherapy + radiotherapy + surgery	18	6.3 ± 4.5	6 (0–17)	0.950
	Chemotherapy + radiotherapy	4	6.0 ± 4.1	4.5 (3–12)	
	Chemotherapy + surgery	3	7.3 ± 9.3	3 (1–18)	
EEH score	Radiotherapy + surgery	35	40.8 ± 3.6	42 (25–47)	
	Chemotherapy + radiotherapy + surgery	18	41.9 ± 2.4	42 (35–45)	0.363
	Chemotherapy + radiotherapy	4	41.5 ± 0.6	41.5 (41–42)	
	Chemotherapy + surgery	3	43.3 ± 2.3	42 (42–46)	

* Kruskal–Wallis non-parametric test, *p* <0.05
